# Structural defects in cilia of the choroid plexus, subfornical organ and ventricular ependyma are associated with ventriculomegaly

**DOI:** 10.1186/2045-8118-9-22

**Published:** 2012-10-09

**Authors:** Ruth E Swiderski, Khristofor Agassandian, Jean L Ross, Kevin Bugge, Martin D Cassell, Charles Yeaman

**Affiliations:** 1 Department of Pediatrics, The University of Iowa, Iowa City, 52242, IA, USA; 2 Department of Anatomy and Cell Biology, The University of Iowa, Iowa City, 52242, IA, USA; 3 Central Microscopy Research Facility, The University of Iowa, Iowa City, 52242, IA, USA

**Keywords:** Bardet-Biedl syndrome, Cilia, Hydrocephalus, Ependyma, Choroid plexus

## Abstract

**Background:**

Hydrocephalus is a heterogeneous disorder with multiple etiologies that are not yet fully understood. Animal models have implicated dysfunctional cilia of the ependyma and choroid plexus in the development of the disorder. In this report, we sought to determine the origin of the ventriculomegaly in four Bardet Biedl syndrome (BBS) mutant mouse strains as models of a ciliopathy.

**Methods:**

Evans Blue dye was injected into the lateral ventricle of wild- type and BBS mutant mice to determine whether obstruction of intra- or extra-ventricular CSF flow contributed to ventriculomegaly. Transmission electron microscopy (TEM) was used to examine the ultrastructure of the choroid plexus, subfornical organ (SFO), subcommisural organ (SCO), and ventricular ependyma to evaluate their ultrastructure and the morphology of their primary and motile cilia.

**Results and discussion:**

No obstruction of intra- or extra-ventricular CSF flow was observed, implying a communicating form of hydrocephalus in BBS mutant mice. TEM analyses of the mutants showed no evidence of choroidal papillomas or breakdown of the blood:CSF barrier. In contrast, structural defects were observed in a subpopulation of cilia lining the choroid plexus, SFO, and ventricular ependyma. These included disruptions of the microtubular structure of the axoneme and the presence of electron-dense vesicular-like material along the ciliary shaft and at the tips of cilia.

**Conclusions:**

Abnormalities in cilia structure and function have the potential to influence ciliary intraflagellar transport (IFT), cilia maintenance, protein trafficking, and regulation of CSF production. Ciliary structural defects are the only consistent pathological features associated with CSF-related structures in BBS mutant mice. These defects are observed from an early age, and may contribute to the underlying pathophysiology of ventriculomegaly.

## Background

Human hydrocephalus is a heterogeneous disorder with multiple etiologies including genetics, developmental defects, viral infection, tumors, hemorrhage and advanced age [1-3]. Congenital hydrocephalus is relatively common and affects 1 in 1,000 live births with a mortality rate of nearly 50% in the absence of shunt placement surgery. Hydrocephalus is characterized by enlarged ventricles resulting from an accumulation of cerebrospinal fluid (CSF) caused by obstruction of intra-ventricular CSF flow (non-communicating hydrocephalus); an imbalance of CSF synthesis and its resorption into the systemic circulation (communicating hydrocephalus), or atrophy of underlying brain tissue or incomplete brain development (hydrocephalus *ex vacuo*) [1].

CSF provides nutritional and metabolic support for the brain, waste removal for the central nervous system, and a protective cushion for the brain and spinal cord. It is produced primarily by epithelial cells of the choroid plexuses of the lateral, third and fourth ventricles and to a lesser degree by the ependyma and parenchyma [4]. Beating of motile cilia on the ependymal lining of the ventricles is thought to facilitate intraventricular CSF circulation, particularly through the narrow aqueduct of Sylvius, as well as increase laminar flow across the ependymal surface [5]. Animal models have implicated damage to or loss of the ependymal layer, reduction in number or loss of its cilia, impaired ependymal cilia motility, dysfunction of the subcommissural organ (SCO), and aqueduct stenosis in the development of hydrocephalus [[2,5]; and references therein]. Further, primary cilia on the apical surface of the choroid plexus epithelium contribute to CSF homeostasis by acting as pressure sensors or as chemosensors that regulate CSF production, osmolarity, or CSF transcytosis from the choroid plexus epithelium into the ventricles via a cilia-based receptor and autonomic system of regulation [6-9].

Despite advances in the study of hydrocephalus, the molecular pathophysiology of this complex disorder, and communicating hydrocephalus in particular is not yet fully understood and requires further investigation. While intra- and extra-ventricular CSF flow in humans and rodents is comparable for the most part, they diverge at the point of resorption through arachnoid granulations. The human brain contains numerous arachnoid granulations while rodents have very few [10] and the choroid plexus plays a role in both the synthesis and resorption of CSF. In rodents, CSF is resorbed through fenestrated capillaries and venules of the choroid plexus that drain into the vein of Galen (vena cerebri interna and vena cerebri magna), in addition to the primary resorption route through the cerebral lymphatic system as well as the spinal cord [10,11]. These differences make the use of animal models such as Bardet Biedl syndrome (BBS) mutant mice valuable tools for the study of non-arachnoid based communicating hydrocephalus and cilia dysfunction.

BBS is a rare autosomal recessive disorder that has become a model for cilia disorders based on a variety of dysfunctional phenotypes associated with the syndrome including retinal degeneration, lack of sperm flagella, obesity, polydactyly, anosmia, learning disabilities, and renal abnormalities [[12,13]; and references therein]. A recent study of 21 BBS patients showed statistically significant increased CSF volume in both the surface of the brain and in the ventricles [14]. BBS is caused by at least 17 genes, which, when individually mutated, give rise to common phenotypes [[13,15-18]; and references therein]. Seven known BBS proteins (BBS1, BBS2, BBS4, BBS5, BBS7, BBS8, BBS9) are components of the BBSome, a coat complex involved in protein trafficking, including receptor trafficking to ciliary and plasma membranes [19-22]. BBS6, BBS10, and BBS12 form part of a chaperone complex required for BBSome assembly, BBS3 recruits the BBSome to the cilia [21,23,24] and BBS17 (Leucine-zipper transcription factor-like 1; LZTFL1) is a negative regulator of BBSome entry into cilia [18,23]. BBS mutant mice have provided valuable insights into the underlying pathophysiology of the disorder by manifesting cardinal features of the human phenotype including obesity, retinal degeneration, male infertility, and olfactory deficits [[25-27]; and references therein].

In a previous report [25], we described a new neuroanatomical phenotype in *Bbs2*^*−/−*^, *Bbs4*^*−/−*,^*Bbs6*^*−/−*^ and *Bbs1*^*M390R/M390R*^ knockin mutant mice (mice homozygous for the most common human BBS mutation that converts a methionine codon to an arginine codon). Each of the three BBS knockout strains as well as the *Bbs1*^*M390R/M390R*^ mice exhibit ventriculomegaly of the lateral and third ventricles of the brain, thinning of the cerebral cortex, and a reduction in the size of the hippocampus and corpus striatum. Unlike severe forms of hydrocephalus observed in other rodent models that result in embryonic or perinatal death or a dome-shaped cranium, the BBS mutant strains used in this study had no distortion of the cranium, the ventriculomegaly was not present at birth, and was progressive in nature [25]. We hypothesized [25] that the ventriculomegaly is caused by atrophy or incomplete development of brain tissue resulting in a compensatory *ex vacuo* enlargement of the ventricles, or that compression of the cerebral cortex, hippocampus and corpus striatum are secondary effects of the enlarged ventricles caused by a yet unknown mechanism. Interestingly, transmission electron microscopy (TEM) analysis showed that a subpopulation of ependymal cilia lining the third ventricle of *Bbs1*^*M390R*/*M390R*^ mice had swollen tips that contained vesicle-like inclusions and electron-dense material. These observations suggested that impaired flow of CSF without disruption of CSF production underlie the observed ventriculomegaly.

In the current report, we sought to investigate the potential contribution of structural defects in cilia of the central nervous system linked to hydrocephalus, particularly those of the choroid plexus, ependyma, SCO and subfornical organ (SFO) to the ventriculomegaly in BBS mutant mice. We examined CSF flow *in vivo* and performed a TEM analysis of tissue morphology and cilia structure of the choroid plexus, SCO, SFO, and ventricular ependyma.

## Methods

### Mice

Wild- type, *Bbs1*^*M390R*/*M390R*^, *Bbs2*^−/−^, *Bbs4*^−/−^, and *Bbs6*^−/−^ mice were generated and maintained as described previously [25]. All studies adhered to guidelines established for the care and use of experimental animals and were approved by the Animal Care and Use Committee of the University of Iowa.

### Visualization of intra-ventricular and extra-ventricular CSF flow

Seven month-old wild- type and *Bbs4*^*−/−*^ mice (n = 3 for each genotype) were anesthetized with a mixture of ketamine (91 mg/kg) and xylazine (9.1 mg/kg) intraperitoneally (i.p.). The fur, skin, membrane and musculature on the surface of the skull were removed and a small hole was drilled into the skull at Bregma level 33. Evans Blue dye (5–10 μl, 2% in 1X PBS; phosphate-buffered saline minus Ca^+2^ and Mg^+2^) was injected slowly into the lateral ventricle using a 25 μl Hamilton syringe to visualize the movement of CSF. The syringe was left in the ventricle post-injection to prevent CSF loss and the dye was allowed to circulate with the CSF for 20 min. Mice were euthanized by an overdose of ketamine and xylazine followed by cervical dislocation and whole mice were immediately frozen at −20°C. The next day, frozen heads were cut in the axial, coronal and sagittal planes and photographed with an Olympus SZX12 stereomicroscope while the tissue was still frozen to prevent dye diffusion.

### TEM

To examine the ultrastructure of ependyma from the lateral and third ventricles and choroid plexuses from the lateral ventricles prior to the onset of ventriculomegaly seen at P9, newborn (P0) and P2 wild- type and *Bbs1*^*M390R*/*M390R*^ animals (n = 3 for each age and genotype) were euthanized and the skin surrounding the skull was removed. The intact skull was placed in 4% paraformaldehyde in 1X PBS for 4–5 hr at 4°C, and transferred to 2.5% glutaraldehyde-0.1 M cacodylate buffer overnight at 4°C. The next day, intact skulls were stabilized in 2% agarose and 100 μm-thick sections were cut with a vibratome, post-fixed with 1% OsO4, rinsed, dehydrated in a series of alcohol and flat embedded in Eponate-12 epoxy resin (Ted Pella, Redding, CA). Tissues were sectioned (85 nm thickness) with a Leica UC-6 ultramicrotome (Wein, Austria). Sections were counterstained with uranyl acetate and lead citrate, and photographed with a JEOL JEM-1230 (Tokyo, Japan) transmission electron microscope. Electron microscopic images were taken with a Gatan UltraScan 1000 (Pleasonton, CA) 2kx2k CCD digital camera.

To study the progression of ventriculomegaly in P9 *Bbs1*^*M390R/M390R*^ and *Bbs2*^*−/−*^, *Bbs4*^*−/−*^ and *Bbs6*^*−/−*^ mice up to 2 years of age (n = 3 for each age and genotype), the protocol was modified to include transcardial perfusion of anesthetized mice with 1X PBS, followed by a solution of 4% paraformaldehyde-0.25% glutaraldehyde. Brains were removed and post-fixed overnight with 2.5% glutaraldehyde-0.1 M cacodylate buffer. The next day, 100 μm-thick sections were cut with a vibratome and processed for TEM analysis of the lateral and third ventricles, choroid plexus, SCO and SFO as described above.

### MRI of brain ventricles

Sagittal MRI images of wild- type and *Bbs1*^*M390R/M390R*^, *Bbs2*^−/−^, *Bbs4*^−/−^, and *Bbs6*^−/−^ mouse brains (n = 3 for wild type and each BBS mutant strain) was performed as described [25]. All images were collected from 6 month-old mice with the exception of *Bbs1*^M90R/M390R^ mice which were 3.5 months old.

## Results

### Intra-ventricular and extra-ventricular CSF flow

**Figure 1:**
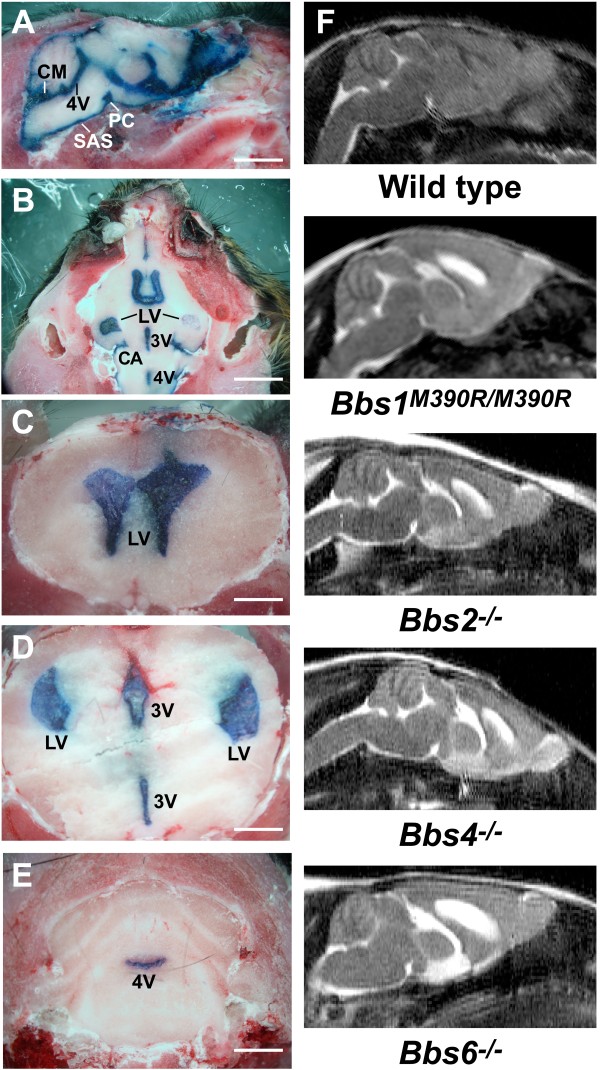
**BBS mutant mice exhibit no obstruction of CSF flow.** Sagittal view of the head of a 7 mo.-old wild- type mouse (**A**) and axial (**B**) and coronal (**C**-**E**) views of *Bbs4*^*−/−*^ mice injected with 2% Evans Blue dye into the lateral ventricle to visualize CSF circulation *in vivo*. *Bbs4*^*−/−*^ mice exhibit no impediment in CSF intra- or extra-ventricular flow as seen by the presence of dye in the enlarged lateral ventricles, third and fourth ventricles, cisterns and subarachnoid space. Representative T-1 weighted sagittal MRIs show no impediment to CSF flow in BBS mutant mice (**F**). All MRI images were collected from 6 month-old mice with the exception of 3.5 month-old *Bbs1*^*M390R/M390R*^ mice. CA (cisterna ambiens), CM (cisterna magna), PC (pontine cistern), SAS (subarachnoid space), 3 V (3^rd^ ventricle), 4 V (4^th^ ventricle). Bars equal 2 mm (**A**, **B**) and 1 mm (**C**-**E**).

To test whether obstruction of intra- or extra-ventricular CSF flow contributed to ventriculomegaly in BBS mutant mice, we visualized *in vivo* CSF flow in 7 month-old wild -type and *Bbs4*^*−/−*^ mice with pronounced enlargement of the lateral and third ventricles. Evans Blue dye was injected into the lateral ventricle of anesthetized mice, allowed to circulate with the CSF throughout the brain for 20 min., following which animals were euthanized. Axial, coronal or sagittal sections from mutant mouse brains revealed no obstruction of intra- or extra-ventricular CSF flow, as seen by the presence of dye in the enlarged lateral ventricles, the third and fourth ventricles, the cisterns, and subarachnoid space (Figure 1A-E). Similar results were observed in comparably aged *Bbs1*^*M390R*/*M390R*^ mice (data not shown). Absence of CSF flow obstruction was further documented in sagittal plane MRIs of 3.5 month-old *Bbs1*^*M390R*/*M390R*^ mice, and 6 month-old *Bbs2*^*−/−*^, *Bbs4*^*−/−*^, and *Bbs6*^*−/−*^ mice (Figure 1F). As reported earlier [25] there is no significant difference in the degree of ventriculomegaly in BBS mutant mice between 3.5-6 months of age. Altogether, these data indicated a communicating form of hydrocephalus in these BBS mutant mouse strains.

### Choroid plexus ultrastructure

**Figure 2:**
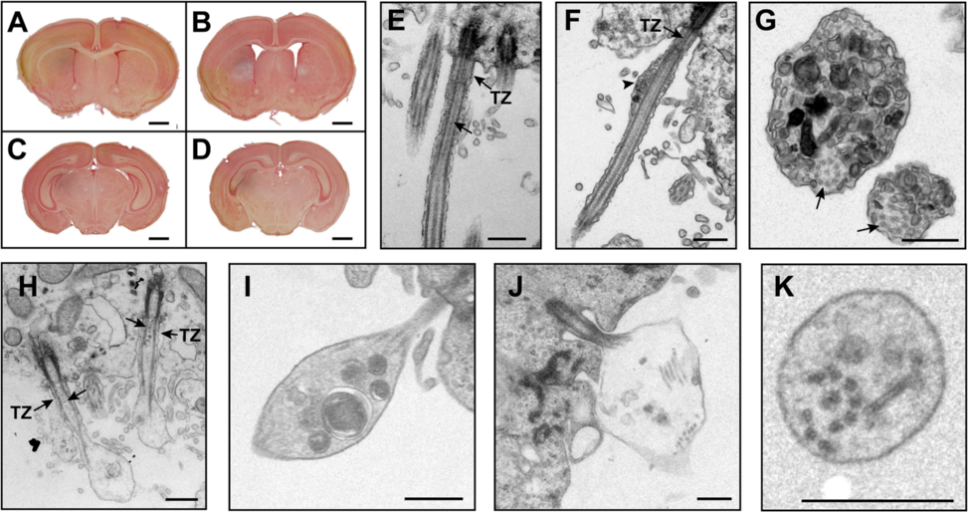
***Bbs1^*M390R/M390R *^*mouse ependymal cilia exhibit structural abnormalities as early as P9.** Neutral red-stained coronal sections of P9 wild-type (**A**, **C**) and *Bbs1*^*M390R/M390R*^ mice (**B**, **D**) illustrate the early appearance of lateral ventricle enlargement while the 3^rd^ ventricle has not yet enlarged. TEM micrographs of P9 wild –type (**E**) and mutant (**F**, **G**) *Bbs1*^*M390R/M390R*^ ependymal cilia, 3.5 week-old (**H**) and 7 month-old (**I**-**K**) *Bbs1*^*M390R/M390R*^ cilia show typical IFT-like particles (arrows) and atypical vesicle-like inclusions and electron dense material (arrowheads) in cross-section and in longitudinal sections. The foreign material appears just distal to the transition zone (**F**) or between the axoneme and the ciliary membrane (**F**, **G**). In some extreme cases, the axoneme appears to be disrupted with bloating of the cilia beyond the transition zone (**H**-**J**). Disruption of the 9 + 2 axoneme is seen in cross-section in addition to the presence of electron-dense material (**K**). Note the broken radial spokes that link the peripheral microtubule doublets to the central pair and the broken nexin links between the peripheral microtubule doublets (**K**). TZ (transition zone), IFT-like particles are noted by arrowheads. Arrows point to axonemes (**G**). Bars equal 1 mm (**A**-**D**), 0.5 μm (**E**-**K**).

**Figure 3:**
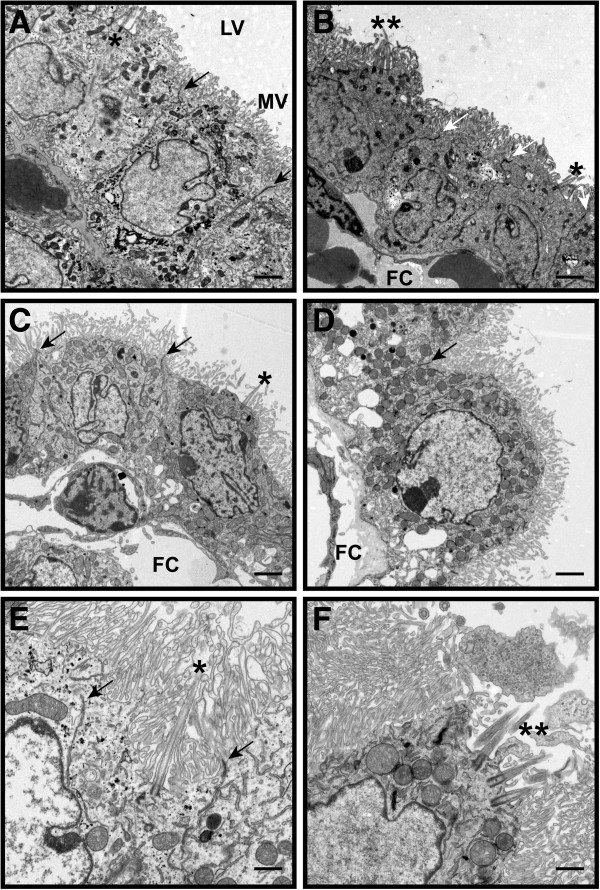
**Choroid plexus epithelial ultrastructure is intact in BBS mutant mice.** TEM micrographs of the choroid plexus epithelium from the lateral ventricles of P0 wild- type (**A**) and *Bbs1*^*M390R/M390R*^ mice (**B**), P9 *Bbs4*^*−/−*^ mice (**C**), and 15 week-old *Bbs6*^*−/−*^ mice (**D**). The epithelial cells appear healthy with numerous microvilli at the apical surface, intact tight junctions between cells, and an intact basolateral membrane, indicating no apparent disruption of the blood-CSF barrier at the ultrastructural level. Single asterisks (*) highlight solitary primary cilia. Double asterisks (**) indicate clusters of cilia. Arrows point to tight junctions between cells. Higher magnification of *Bbs4*^*−/−*^ choroid plexus solitary cilia (**E**) and *Bbs6*^*−/−*^ clustered cilia (**F**). FC (fenestrated capillary), LV (lateral ventricle), MV (microvilli). Bars equal 2 μm in A-D and 1 μm in E, F.

CSF is formed by the net directional transport of sodium, chloride, bicarbonate and water from the rich choroidal blood supply across the choroid plexus epithelium via basal membrane transporters and apical membrane ion channels and aquaporin water channels into the ventricles [28]. Integrity of the choroid plexus epithelium, therefore, is critical for maintaining the blood:CSF barrier and for CSF homeostasis. To determine whether choroid plexus ultrastructure is affected in BBS mutant mice, we used TEM to examine the brains of newborn (P0) wild- type and BBS mice and during the early stages of ventriculomegaly at P9 (the earliest stage other than P0 used in the study) (Figure 2A-D) and during its progression at 15 weeks. At birth, there was no evidence of ventriculomegaly (data not shown) and the *Bbs1*^*M390R*/*M390R*^ choroid plexus epithelium appeared normal and contained numerous microvilli together with solitary primary cilia and clusters of primary cilia on the apical membrane surface of some of the cells (Figure 3 A,B). Tight intracellular junctions of the apical epithelium were intact, as were the basolateral membranes. Comparable results were observed in 15 week-old *Bbs4*^−/−^ and *Bbs6*^*−/−*^ mice (Figure 3C-F). Based on these findings, there appeared to be no gross disruption of the blood:CSF barrier at the ultrastructural level.

### Ultrastructural analysis of BBS mutant choroid plexus cilia

**Figure 4:**
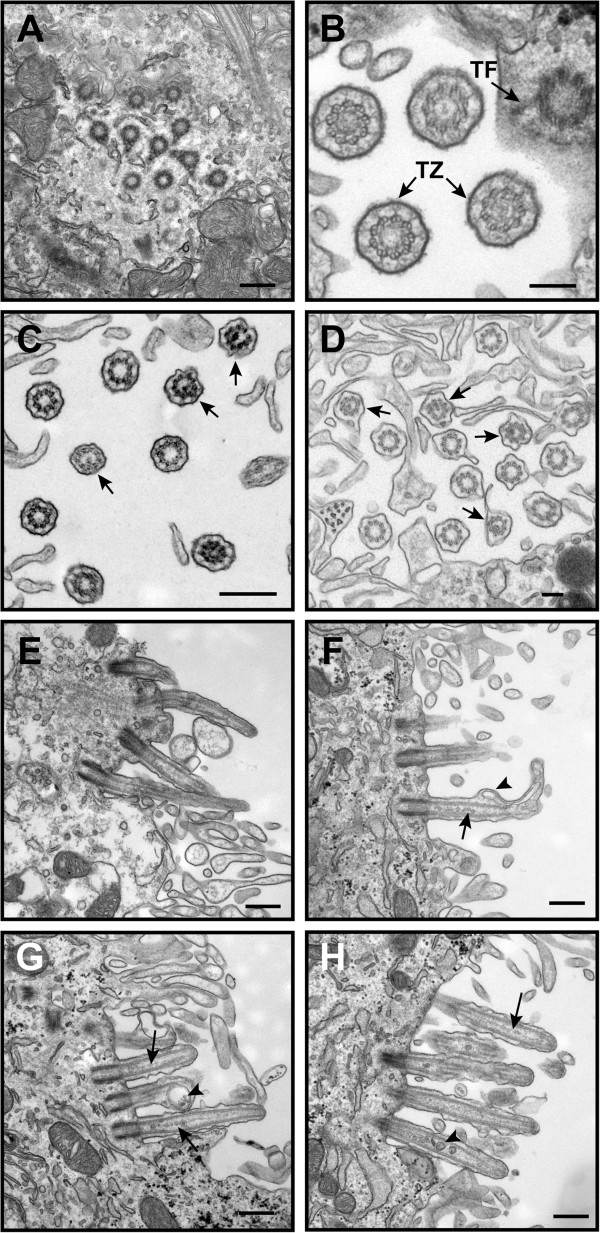
**BBS mutant mice exhibit structural defects in choroid plexus epithelial cilia.** TEM micrographs show normal multiple basal bodies and basal feet in P9 *Bbs1*^*M390R/M390R*^ mouse choroid plexus cilia clusters (**A**). P0 *Bbs1*^*M390R/M390R*^ cilia have intact Y-links of the ciliary necklace that link the microtubule doublets and the ciliary membrane in the region of the transition zone (TZ) of the ciliary necklace between the basal body and axoneme (**B**). Transition fibers (TF) are also seen connecting the basal body to the epithelial plasma membrane. Fifteen month-old *Bbs6*^*−/−*^ primary cilia clusters show typical variations of the 9 + 0 mictrotubule arrangement of the axoneme distal to the basal body (arrows; **C**, **D**). Longitudinal TEM micrographs of choroid plexus cilia clusters from P0 wild- type (**E**) and *Bbs1*^*M390R/M390R*^ mice (**F**), P2 *Bbs1* mutant mice (**G**), and P10 *Bbs1* mutant mice (H) show disruption along the elongating ciliary axoneme as well as vesicle-like inclusions and electron-dense material that are larger than IFT particles along the ciliary shaft in a sub-population of cilia. In some cilia, the electron-dense material appears between the ciliary plasma membrane and the axoneme (**F**, **G**) while in others it is found in the lumen of the cilium, surrounded by the peripheral microtubule doublets. Arrows point to IFT-like particles (**F**-**H**). Arrowheads point to large electron-dense, vesicle-like material (**F**-**H**). (Bars, 0.5 μm (**A**), 0.2 μm (**B**), 0.5 μm (**C**), 0.2 μm (**D**), 0.5 μm (**E**-**H**).

In light of the growing body of evidence that choroid plexus primary cilia play an important role in signaling and CSF homeostasis [7-9], we used TEM to examine their ultrastructure in young (P0 and P9) BBS mutant mice as well as in 15 month-old mice when ventriculomegaly had progressed. Seen in cross-section, the choroid plexus epithelium of *Bbs1*^*M390R/M390R*^ mice had multiple intact basal bodies (Figure 4Ac). The Y-shaped links of the ciliary necklace that connect the microtubule doublets to the ciliary membrane in the transition zone; the region of the basal body where the triplet microtubules transition to the doublet microtubules of the axoneme appeared to be intact in the P0 mutant mice (Figure 4B). Compared to the abundant ependymal cilia lining the ventricles, primary cilia on the apical surface of the choroid plexus were rare and occurred in clusters. Examination of cross-sections of 15 month-old wild- type and *Bbs6*^*−/−*^ mouse choroid plexus cilia along the ciliary shaft distal to the transition zone showed occasional clusters of primary cilia with typical derivatives of the 9 + 0 arrangement of microtubule doublets including 8 + 1 axonemes with displacement of one pair of peripheral doublets, and rarely, 7 + 2 axonemes with displacement of 2 pairs of peripheral doublet (Figure 4C, D) in agreement with previous studies [7,9,29,30].

When clusters of choroid plexus cilia from P0, P2, and P10 mutant animals were viewed longitudinally, a sub-population of cilia contained vesicle-like inclusions and electron-dense material along the ciliary shaft distal to the transition zone. This material was larger than the IFT-like particles typically seen in the choroid plexus cilia (Figure 4F-H). The mutant cilia also exhibited disruption of the ciliary axoneme that was not seen in comparably aged wild- type mice (Figure 4 E-H). In some cilia, the electron-dense material was found between the peripheral microtubule doublets of the axoneme and the ciliary plasma membrane where IFT particles are typically located (Figure 4F, G), while in others it was found in the ciliary lumen, surrounded by the peripheral microtubule doublets (Figure 4H).

### Ultrastructural analysis of BBS mutant ventricular ependymal cilia

**Figure 5:**
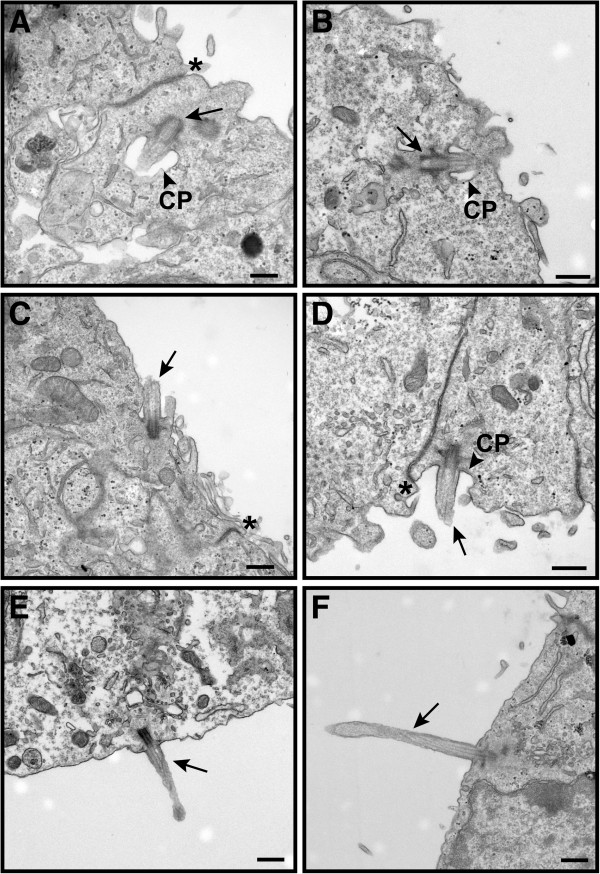
**Genesis of ependymal cell primary cilia appears normal in P0 *Bbs1^*M390R/M390R *^*mice.** TEM micrographs of P0 wild- type (**A**, **C**, **E**) and *Bbs1*^*M390R/M390R*^ mice (**B**, **D**, **F**) show normal stages of primary cilia formation in the lateral ventricle. The mother centriole (arrow) is surrounded by the secondary centriole and approaches the ependymal plasma membrane (**A**, **B**). The cilium (arrow) then docks and fuses with the ependymal plasma membrane (**C**, **D**) and elongates via incoming secondary ciliary vesicles and intraflagellar transport (**E**, **F**). The ciliary pocket (CP) is evident in panels **A**, **B**, and **D** (arrowhead). The ependyma appears healthy and exhibits tight junctions between cells (*). Bars equal 0.2 μm (**A**) and 0.5 μm (**B**-**F**).

The structural abnormalities observed in some choroid plexus cilia as early as the time of birth prompted us to examine more closely the ependymal cilia of BBS mutant mice during the progression of ventriculomegaly. Similar to earlier studies [31], we observed in *Bbs1*^*M390R/M390R*^ newborn mice the normal stages of primary ciliogenesis in the radial glia progenitor cells that subsequently transform into ventricular ependymal cells: (1) the mother centriole surrounded by the secondary centriolar vesicle, forming the ciliary pocket (Figure 5A, B); (2) docking of the mother centriole at the apical cell surface and fusion of the ciliary membrane with the ependymal plasma membrane (Figure 5C, D) and (3) elongation of the mature centriole (basal body) to form the cilium with the help of secondary ciliary vesicles and intraflagellar transport (IFT) (Figure 5E, F). Based on these observations, the BBS mutant ependymal primary cilia appeared to have assembled correctly.

**Figure 6:**
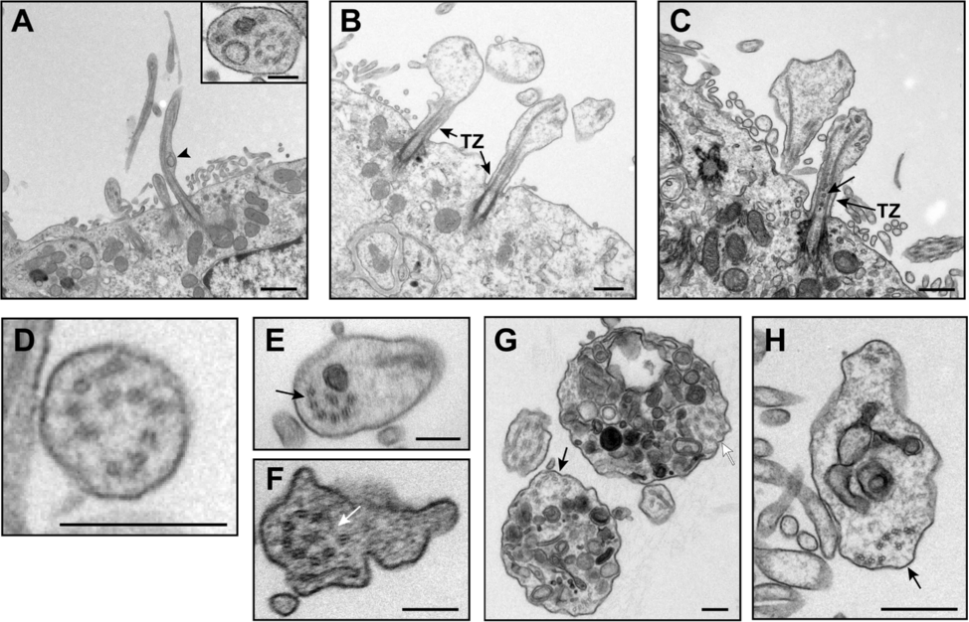
**Structural abnormalities are present in the ependymal cilia of *Bbs2***^***−/−***^**, *Bbs4***^***−/−***^**, and *Bbs6***^***−/−***^**mice.** TEM micrographs illustrate the presence of vesicular-like inclusions and electron-dense material in a sub-population of BBS mutant mouse ependymal cilia lining the enlarged lateral ventricles (**A**,**B**, **D**-**F**) and 3^rd^ ventricles (**C**, **G**, **H**) in 4 month-old *Bbs2*^*−/−*^ mice (**A**, **D**), 2 month-old *Bbs4*^*−/−*^ mice (**B**, **E**, **F**) and 8 month-old *Bbs6*^*−/−*^ mice (**C**, **G**, **H**). In some cilia, these structural abnormalities appear slightly distal to the transition zone between the basal body and the ciliary axoneme (A) while in others bloating of cilia is observed (**B**, **C**). Cross-sections illustrate disruption of the 9 + 2 axoneme in a subpopulation of cilia. Note the broken radial spokes that attach the peripheral microtubule doublets to the central pair, and the broken nexin links that connect the peripheral microtubule doublets (**D**-**H**). Arrows point to disrupted axonemes in E, F, G (lower region), and H. Typical IFT-like particles are denoted by an arrow in (**C**). TZ (transition zone). Bars, 1 mm (**A**-**D**), 0.5 μm (**E**-**L**).

In mice, ependymal primary cilia are replaced by multiple motile cilia during the second week of postnatal life [32,33]. As early as P9, we observed ultrastructural ciliary defects in the *Bbs1*^*M390R/M390R*^ motile cilia and modest enlargement of the lateral ventricles (Figure 2A-D). We observed vesicle-like inclusions and electron-dense material along the ciliary shaft distal to the transition zone in approximately 20% of ependymal cilia lining the lateral and 3^rd^ ventricles. These structural abnormalities were also seen in mutant mice up to 2 years of age, but not in their wild-type littermates. Small IFT-like particles [34] were seen in the transition zone and proximal axoneme of wild-type cilia (Figure 2E) as well as in some BBS mutant cilia (Figure 2H). In mutant cilia, electron-dense material larger than ITF-like particles was frequently observed just distal to the transition zone between the microtubules and the ciliary membrane where IFT particles were typically found (Figure 2F), while in other cilia this material was located more distally along the ciliary shaft (Figure 2G). In some extreme cases, the region just beyond the transition zone appeared to be bloated and filled with electron-dense vesicular material (Figure 2 H-J). Seen in cross-section, the presence of electron-dense material coincides with a disrupted axoneme in some cilia (Figure 2K) while in others the axoneme appeared to be intact (Figure 2G). We observed similar structural abnormalities in the ependymal cilia lining the lateral and third ventricles of 2 to 7 month-old *Bbs2*^*−/−*^, *Bbs4*^*−/−*^*,* and *Bbs6*^*−/−*^ mice (Figure 6A-H).

**Figure 7:**
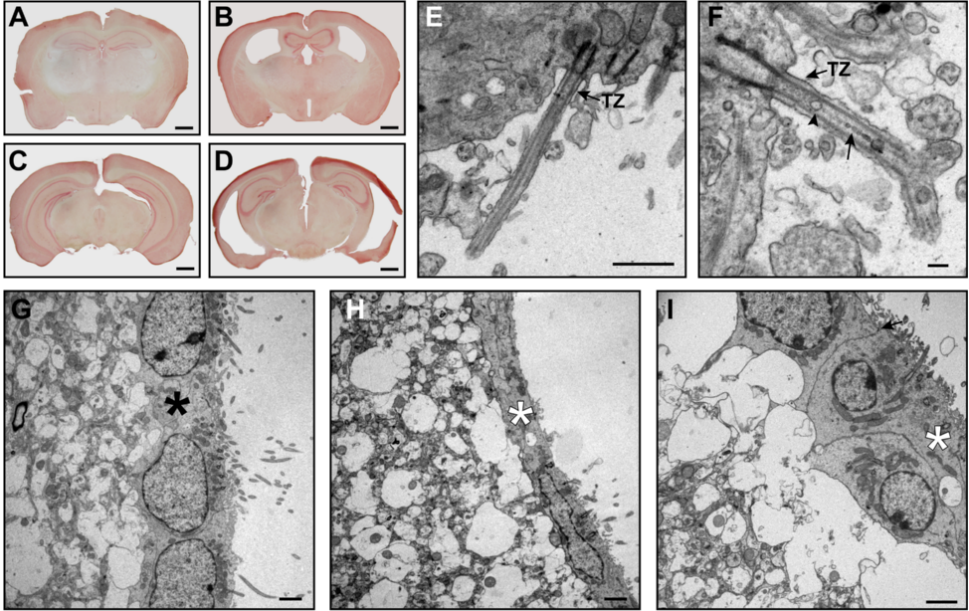
**Two-year old *Bbs4^*−/− *^*mouse lateral ventricles exhibit ependymal thinning and vacuolization of the neuropil.** Neutral red stained coronal sections of 2 yr.-old wild- type (**A**, **C**) and *Bbs4*^*−/−*^ brains (**B**, **D**) illustrate the progressive nature of the lateral and third ventricle enlargement. TEM micrographs of ependymal cilia of the lateral ventricles of wild- type (**E**) and *Bbs4*^*−/−*^ mice (**F**) show reduced numbers of cilia in the mutant mice as well as a mixture of normal and abnormal cilia. Note the presence of typical IFT-like particles and larger vesicle-like material in some *Bbs4*^*−/−*^ cilia (**F**). Thinning of the ependymal layer of *Bbs4*^*−/−*^ mice (**H**) is apparent in some regions of the ependyma (**G**) compared to wild-type mice. Other regions of the ependyma look healthy with intact zona adherens junctions between cells (arrow) but have large vacuoles in the underlying neuropil (**I**). Asterisks denote the ependymal layer. Bars 1 mm (**A**-**D**), 1 μm (**E**), 0.2 μm (**F**), 2 μm (**G**-**I**).

By 2 years of age, the extent of ventriculomegaly had progressed in BBS mutant mice (Figure 7A-D). At this age, wild- type ependymal cells looked normal and had numerous motile cilia (Figure 7E, G) while the ependymal layer of *Bbs4*^*−/−*^ mutant mice was significantly thinner in some regions (Figure 7H). Other areas of ependyma appeared normal with intact zonae adherentes between cells, yet the underlying neuropil showed evidence of edema (Figure 7H, I). The BBS mutant ependyma had very few microvilli or cilia on its surface and some cilia appeared structurally abnormal (Figure 7F). Comparable changes were also observed in 2 year-old *Bbs1*^*M390R/M390R*^ mice (data not shown).

### Ultrastructural analysis of BBS mutant SCO and SFO

**Figure 8:**
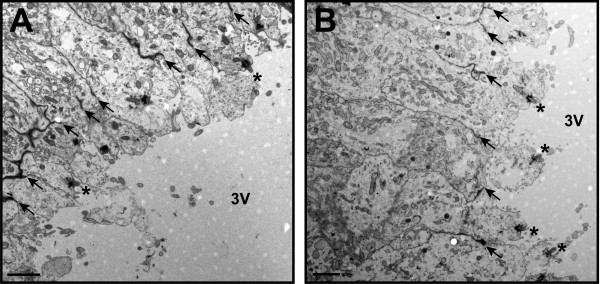
**BBS mutant mouse SCO ultrastructure.** TEM micrographs of coronal sections of 3.5 week-old wild-type (**A**) and *Bbs1*^*M390R/M390R*^ (**B**) SCO show typical goblet-shaped ependymal cells with intact apical zona adherens junctions between cells (arrows). Single cilia are found on ependymal cell protrusions (arrowheads). The motile cilia seen in cross-section in the lumen of the third ventricle may represent cilia from other SCO cells or from adjacent ventricular ependymal cells.

No gross malformations in the SCO of *Bbs1*^*M390R/M390R*^ mice compared to their wild-type littermates were observed (Figure 8). The apical zonae adherentes between cells appeared to be intact. Single cilia were identified on the apical protrusions of the goblet-shaped ependymal cells that project into the lumen of the third ventricle and are in contact with CSF. Previous studies have shown that these cilia have 9 + 2 axonemes [35,36]. In light of the apparent intact SCO ultrastructure and the patency of the aqueduct of Sylvius and fourth ventricle in BBS mutant mice, it is unlikely that abnormalities in the SCO contribute to ventriculomegaly yet we cannot rule out the possibility of absent or defective Reissner’s fibers**.**

**Figure 9:**
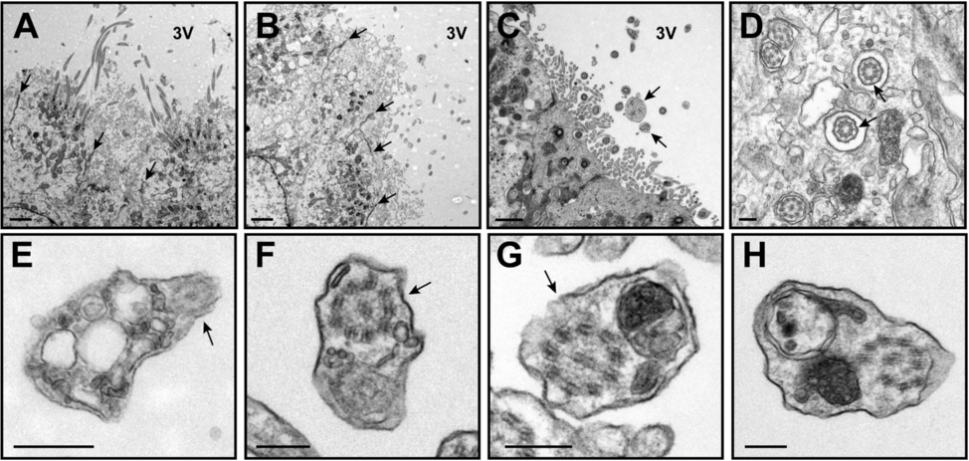
**BBS mutant mouse SFO ultrastructure.** TEM micrographs of coronal sections of 3.5 wk-old wild-type (**A**), *Bbs1*^*M390R/M390R*^ (**B**) and *Bbs6*^*−/−*^ SFO show an intact ependymal layer with zona adherens junctions between cells and numerous motile cilia on the apical surface. Transition zones (arrows in **C**) appear normal in 8 mo.-old *Bbs6*^*−/−*^ mice. Electron-dense and vesicle-like material is seen in *Bbs1*^*M390R/M390R*^ and *Bbs6*^*−/−*^ SFO cilia in the presence (**G**) and absence (**D**-**F**) of axonemal disruption. Arrows point to axonemes. Bars equal 2 μm (**A**, **B**,**C**) and 0.2 μm (**D**-**H**).

We did not observe any gross morphological changes in the cellular ultrastructure of the SFO in wild-type, *Bbs1*^*M390R/M390R*^ or *Bbs6*^*−/−*^ mutant mice and the apical zonae adherentes between cells appeared to be intact (Figure 9A, B). In the coronal plane, the ventral-most region of the ependymal surface of the SFO that faces the lumen of the third ventricle was sparsely ciliated in both wild-type and mutant mice, in agreement with previous studies [37,38]. The transition zone at the base of the SFO cilia appeared to be intact in BBS mutant mice. However, axonemal disruption and the presence of electron-dense vesicle-like material along the ciliary shaft was observed in a sub-population of motile SFO cilia in BBS mutant mice similar to that seen in the cilia of the choroid plexus and ventricular ependyma of these mice (Figure 9C-H).

## Discussion and conclusion

Animal models of hydrocephalus have demonstrated a relationship between cilia defects of the choroid plexus and ependyma and the development of the disorder [[2,5,7-9,25,33,39-41] and references therein]. In this report, we used functional assays and ultrastructural analyses to examine ventriculomegaly in four BBS mutant mouse strains widely used as models of ciliopathies. We hypothesized that the ventriculomegaly in BBS mice might be due to ultrastructural damage to the epithelia or cilia of the choroid plexus, ependyma, SCO, or SFO. We demonstrated that a subpopulation of primary and motile cilia in the choroid plexus, SFO and the ependymal lining of the lateral and third ventricles had axonemal defects and in some instances contained electron-dense vesicle-like material along the ciliary shaft and at the tips of cilia. Notably, the mutant mice exhibited no physical obstruction of intra- or extra-ventricular flow implying the ventriculomegaly is associated with a communicating form of hydrocephalus.

It is possible that CSF resorption through the choroid plexus vasculature into the vein of Galen in the BBS mutant animals may be physically impaired due to compression of the vein as a secondary effect of ventricular enlargement or that an impediment of CSF flow across the cribriform plate into the lymphatics of the olfactory turbinates could lead to hydrocephalus [42]**.** These obstructions could exacerbate the phenotype and will require further examination.

### Ventriculomegaly due to increased CSF volume

The most common causes of communicating hydrocephalus in humans are increased CSF synthesis due to choroid plexus papillomas and impaired CSF resorption by the arachnoid granulations [4]. To evaluate possible choroidal anomalies, we analyzed the ultrastructure of the choroid plexuses of the lateral and third ventricles in BBS mutant mice using TEM and found no evidence of choroidal papillomas. The normal appearance of the choroid plexus epithelia with intact junctions between cells argues against the possibility of passive diffusion of molecules from the choroid plexus vasculature into the CSF that could have led to breakdown of the blood:CSF barrier and a loss of CSF homeostasis. Still, we cannot rule out the possibility that the mice produce an increased volume of CSF as a compensatory response to hydrocephalus *ex vacuo* due to atrophy of underlying brain tissue or incomplete brain development. Future studies of the temporal appearance of ventriculomegaly by MRI analysis in conjunction with cell proliferation and cell death assays will address this issue. Another avenue of future research would be to examine the presence and localization of the aquaporin channels in the choroid plexus that control water transfer [[43]; and references therein].

### Ventriculomegaly and impaired cilia function in the SCO and SFO

Dysfunction of the SCO, a specialized zone of ependyma located on the roof of the third ventricle at the entrance to the aqueduct of Sylvius, has been shown to result in hydrocephalus in a number of animal models [44]. The ependymal cells of the SCO secrete glycoproteins that aggregate to form the threadlike Reissner’s fibers that maintain patency of the aqueduct and central canal of the spinal cord [5,44]. In the absence of a correctly functioning SCO, patency of the aqueduct is compromised, leading to aqueductal stenosis and non-communicating hydrocephalus [45]. Given the apparent intact SCO ultrastructure and patency of the aqueduct of Sylvius and fourth ventricle in BBS mutant mice as old as 2 years of age, it appears unlikely that abnormalities in the SCO contribute to ventriculomegaly. Further, aqueductal stenosis does not appear to be a secondary effect of ventriculomegaly in the BBS mutant mice. Still, the lack of evidence to support the presence of Reissner’s fibers in the mutant mice remains to be addressed.

The SFO, which protrudes into the midline anterior wall of the third ventricle at the junction of the intraventricular foramina of Munro, is anatomically well positioned for its role in osmosensation and the regulation of body and CNS water balance. Although there is currently no known role for the primary and motile cilia that line the SFO [37,38], we noted structural defects in some of these cilia in BBS mutant mice. They exhibited the axonemal abnormalities and electron-dense vesicle-like material as seen in primary cilia of the choroid plexus and motile ventricular ependymal cilia.

### Defective cilia maintenance

BBS has been described as a degenerative disease of the cilium in which certain proteins accumulate in the cilia over time, leading to progressive ciliary dysfunction [46]. Signaling proteins have been shown to accumulate in BBS mutant *Chlamydomonas reinhartii* flagella [46] and in zebrafish the absence of BBS proteins leads to delayed retrograde transport [47,48]. The BBSome functions in the trafficking of membrane proteins between the plasma and ciliary membranes. Several G-protein coupled receptors including MCHR, SSTR3 and dopamine receptor 1 accumulate abnormally within neuronal cilia of BBS mutant mice [49,50] and ciliary trafficking of the hedgehog signal transducer Smoothened is controlled by the BBSome [18,22,51]. Recently, the BBSome has been shown to control IFT assembly and IFT turnaround at the ciliary tip [52].

It is possible that the electron-dense vesicle-like material found in BBS mutant mouse choroid plexus, SFO and ependymal cilia are the result of defective cilia maintenance. The dimensions of the electron-dense, vesicle-like material are too large to have entered the cilia though the transition zone [53]. A more likely scenario is that the material accumulates in the cilia due to defective anterograde and/or retrograde IFT caused by damage to the axoneme that has been observed in some of the defective cilia or that turnaround at the ciliary tip is impaired in the mutants [52].

The presence, at birth, of electron dense vesicle-like inclusions in some of the BBS mutant mouse choroid plexus primary cilia may result from defective ciliary maintenance could lead to impaired signaling between the choroid plexus cilia and epithelium and cause an ionic imbalance in the CSF resulting in its overproduction. The choroid plexuses develop during the early stages of mammalian embryogenesis and are fully formed, ciliated and functional at birth [54]. Interestingly, studies of the Tg^737orpk^ mouse and *in vitro* primary cultures of choroid plexus epithelia have underscored the importance of the choroid plexus and its primary cilia in the regulation of CSF production. It has been proposed that protein mislocalization caused by defective IFT in the Tg^737orpk^ mouse choroid plexus primary cilia impairs their ability to signal to the underlying epithelium via a cAMP-regulated mechanism in order to modulate CSF production [7,8]. Furthermore, clusters of primary cilia present on the apical surface of porcine choroid plexus primary epithelial cell cultures have been shown to act as negative regulators of fluid transcytosis by decreasing intracellular cAMP levels. These cilia also express neuropeptide FF (NPFF) receptor 2 thought to play a role in chemosensory function and regulation of fluid transport [9].

There has been a resurgence of interest in the study of higher vertebrates for the role of motile cilia in chemosensation or mechanosensation, functions thought to be ascribed solely to primary, immotile cilia [55-58] following the initial studies in *Paramecium* and *Chlamydomonas* reviewed in [55]. Taste receptors have been localized in the ciliary membranes of mouse tracheal epithelial cells [59], progesterone receptors are present in the motile cilia of the mouse oviduct [60], and the Px2 receptor has been localized in rabbit tracheal motile cilia [61]. The appearance of structurally defective ependymal cilia in BBS mutant mice as early as P9, which is shortly after the replacement of primary cilia by motile cilia in mice [32,33], may impair a sensory mechanism that is relayed to the choroid plexus to regulate CSF synthesis, resulting in a loss of CSF homeostasis and ventriculomegaly.

In conclusion, abnormalities in BBS mutant mouse cilia structure and function have the potential to influence ciliary intraflagellar transport (IFT), ciliary beat frequency, cilia maintenance, protein trafficking, and regulation of CSF production. Ciliary structural defects are the only consistent pathological features associated with CSF-related structures in BBS mutant mice. These defects are observed from an early age, and may contribute to the underlying pathophysiology of ventriculomegaly. Additional research is necessary to establish a causal relationship between the ciliary abnormalities and the development of ventriculomegaly in BBS mutant mice. Continued study of the these mice will add to the growing body of knowledge of the roles played by cilia of the choroid plexus, SFO, and ventricular ependyma in CSF homeostasis as well as in understanding the underlying pathophysiologies of BBS.

## Competing interests

None of the authors have any competing interests.

## Authors’ contributions

RES, KA and MDC conceived and designed the experiments. RES, KA, JLR and KB carried out the experiments. RES, MDC and CY were involved in writing and editing the manuscript and preparation of figures. All authors have read and approved the final version of the manuscript.
